# Body Mass Index and Inguinal Hernia: An Observational Study Focusing on the Association of Inguinal Hernia With Body Mass Index

**DOI:** 10.7759/cureus.11426

**Published:** 2020-11-10

**Authors:** Rekha Melwani, Sadaf Jabeen Malik, Dharmoon Arija, Ihsanullah Sial, Ajay Kumar Bajaj, Adnan Anwar, Atif A Hashmi

**Affiliations:** 1 General Surgery, Al-Tibri Medical College and Hospital, Isra University, Karachi, PAK; 2 General Surgery, Ghulam Muhammad Mahar Medical College, Sukkur, PAK; 3 Physiology, Al-Tibri Medical College and Hospital, Isra university, Karachi, PAK; 4 Pathology, Liaquat National Hospital and Medical College, Karachi, PAK

**Keywords:** inguinal hernia, gender, intra abdominal pressure, body mass index

## Abstract

Objective

An inguinal hernia is a common condition associated with advanced age, male gender, smoking, connective tissue disorder, and factors responsible for increased intra-abdominal pressure. This study aimed to observe the relationship of body mass index with the development of inguinal hernia in males and females.

Methodology

This cross-sectional descriptive study using a non-probability convenient sampling technique was carried out at Al-Tibri medical college and hospital, Karachi, Pakistan. A total of 82 patients were selected: 78 males and four females. The ethical approval for the study was taken from Institutional Research and Ethical Committee. Inclusion criteria based on the patient age above 40 of either gender with complaints of pain in the groin region with clinical findings like swelling and tenderness. Data were analyzed using Statistical Package for the Social Sciences (SPSS) version 20.0 (IBM Inc., Armonk, USA).

Results

The mean age of 82 patients diagnosed with an inguinal hernia on a clinical basis was 47.41 ± 15.49 years. The mean height was 67.09 ± 3.95 inches. The mean weight was 63.5 ± 6.77 kg. The mean BMI was 22.07 ± 2.17 kg/m^2^. Seventy-eight (96.06%) were males, and four (5.9%) were females. Thirty-four (41.5%) patients were diagnosed with right inguinal hernia, 34 (41.5%) - with a left inguinal hernia, and 14 (17.1%) - with a bilateral inguinal hernia. BMI was normal in 68 (86.3%) and low in 14 (20.55%) patients. Our study indicated that patients with normal BMI were more likely to suffer from inguinal hernia than patients with low BMI.

Conclusion

This study concluded that the normal body mass index was associated with a high occurrence of inguinal hernia among the genders. The normal body mass index in males exhibits more inguinal hernia chances than a low body mass index. It was observed that the frequency of unilateral right inguinal hernia is higher than bilateral. Similarly, males are more affected than females.

## Introduction

A hernia is described as a condition where an organ or part of an organ gets displaced and protrudes through the cavity wall containing it [1]. Hernia might be of congenital origin or occur due to the failure of complete development of some structures right after birth or might appear in later life due to obesity, weakness of muscles, any operation, or disease. Parts that are common sites of herniation include umbilicus, groin, linea alba, diaphragm, semi-luna line of Spieghel, and surgical incision. Hernias of the abdominal wall are very commonly observed, accounting for around 15-18% of all surgeries.

Occurrences of inguinal hernias (IHs) are widespread globally [2]. The exact incidence of IH is unknown, but almost 800,000 are repaired every year in the USA alone, and it is calculated that about one in every two men requires repair of an inguinal hernia (IH) in his lifetime [3]. The most frequently observed abdominal wall hernia is IH amounting to approximately 75% of all hernias, including lifetime risk of hernia in 27% of men and 3% in women [4]. Advanced age, male gender, smoking, family history, connective tissue diseases, and increased intra-abdominal pressure have all been factors responsible for developing an IH. However, the relationship between IH development and body weight is still unclear [5, 6].

Symptoms of an inguinal hernia include the feeling of sharp pain on cough, exercise, burning sensations, pain and swelling in the groin region, fullness or heavy sensation in the groin, and scrotal swelling amongst men.

Risk factors responsible for developing an inguinal hernia include male gender and old age, simultaneous systemic connective tissue disorder, or low body mass index (BMI) [7, 8]. However, high BMI increases intra-abdominal pressure because of obesity, leading to the development of IH [9]. Smoking is also presumed to be a risk factor for developing a hernia, but it is uncertain [10]. Intra-abdominal pressure increases as a result of coughing, jumping, etc. and also increases the chance of inguinal hernia [11].

After a physical examination, additional investigation may include imaging tests such as ultrasonography, computed tomography (CT), or magnetic resonance imaging (MRI). These are the diagnostic tools that help in diagnosis [12].

Elective surgery has been recommended for all types of inguinal hernias instead of prescribing medicines because medicines alone cannot cure the disease regardless of the risk of complications such as incarceration or strangulation, so it needs to repair early. Numerous different types of open and laparoscopic hernia surgery are accessible [13]. The objectives of the current research were to observe relationships and find out the association of IH with different BMI categories.

## Materials and methods

This cross-sectional descriptive study using a non-probability convenient sampling technique was conducted at Al-Tibri Medical College and Hospital, Karachi, Pakistan. After attaining ethical approval from the Institutional Research and Ethical Committee, the study was carried out. Informed consent was sought from patients and guardians. A total of 82 patients were included in this research.

Inclusion criteria included patient aged above 40 of either gender with complained of pain in the groin region with clinical findings like swelling and tenderness, cause of strain if present, chronic cough, systemic connective tissue disorder; factors that raise the intraabdominal pressure like constipation, being overweight and the labor that lifts heavy weights, were also included in this study. However, patients with muscular groin pain without swelling or any other evidence of raised intra-abdominal pressure were excluded from the study. Age, height, weight, and BMI were recorded. BMI less than 18.5 kg/m^2 ^and from 18.5 to 24.9 was considered low and normal.

Data analysis was done using Statistical Package for the Social Sciences (SPSS) version 20 (IBM Corporation, Armonk, US). The analysis consisted of calculating means and standard deviations for quantitative data and frequency and percentages for qualitative data.

## Results

Table 1 shows that the mean age of 82 patients suffering from an inguinal hernia was 47.41 ± 15.49 years (p=0.001). The mean height was 67.09 ± 3.95 inches (p=0.034). The mean weight was 63.5 ± 6.77 kg (p=<0.001). The mean BMI was 22.07 ± 2.17 kg/m^2^ (p≤0.001). All the p-values for age, height, and weight are statistically significant (less than 0.05), indicating that BMI affects the incidence of inguinal hernia. There were 78 (96.06%) males and four (5.9%) females in the sample. Thirty-four (41.5%) patients were diagnosed with a right inguinal hernia, 34 (41.5%) with a left inguinal hernia, and 14 (17.1%) with a bilateral inguinal hernia. BMI was normal in 68 (86.3%) and low in 14 (20.55%) patients. This indicates that in our study, patients with normal BMI are more likely to suffer from inguinal hernia compared to patients with low BMI.

**Table 1:** General description of the patients of inguinal hernia SD - standard deviation

Variable	Right inguinal hernia (mean ± SD)	Left inguinal hernia (mean ± SD)	Bilateral inguinal hernia (mean ± SD)	p-value
Age (years)	51.00 ± 17.51	37.82 ± 16.46	53.42 ± 12.50	0.001
Height (inches)	65.64 ± 3.13	67.64 ± 3.30	68.0 ± 5.434	0.034
Weight (kg)	60.94 ± 8.37	59.52 ± 8.53	70.0 ± 3.419	<0.001
BMI (kgm^2^)	21.29 ± 2.30	20.63 ± 2.30	24.29 ± 1.91	<0.001

Table 2 shows that in the right inguinal hernia (RIH) group, the predominant gender was males - 32 (94.1%), in the left inguinal hernia (LIH) group - 32 (94.1%), and in the bilateral inguinal hernia (BIH) group - 14 (100.0%). There were two (5.9%) females in the RIH group, two (5.9%) in the LIH group (p=0.649), with no female in the BIH group. The right side was predominant over the left. BMI was normal in 28 (82.4%) patients in the RIH group, 26 (76.5%) patients in the LIH group, and 14 (100.0%) patients in the BIH group. MBI was low in six (17.6%) patients in the RIH group, in eight (23.5%) patients in the LIH group, and no patients with low BMI were found in the BIH group (p=0.143).

**Table 2:** The frequency of the type of hernia based on gender and BMI BMI - body mass index

Variable		Right inguinal hernia (%)	Left inguinal hernia (%)	Bilateral inguinal hernia (%)	p-value
Gender	Male	32 (94.1%)	32 (94.1%)	14 (100.0%)	0.649
	Female	2 (5.9%)	2 (5.9%)	0 (0.00%)	
BMI	Normal	28 (82.4%)	26 (76.5%)	14 (100.0%)	0.143
	Low	6 (17.6%)	8 (23.5%)	0 (0.00%)	

## Discussion

Hypothetically, obesity is thought to be responsible for the rising frequency of inguinal hernia by elevating abdominal pressure. On the other hand, the risk of developing an inguinal hernia is reported to decline among overweight or obese individuals in the majority of studies. Rosemar et al. have analyzed that in male patients between 47-55 years having an inguinal hernia, a rise of one unit (from three to four kg) BMI, risk of hernia reduces by 4% [14]. Compared to individuals with normal weight, the risk of developing an inguinal hernia is reduced to 43% in obese patients. Additionally, among adult females, obesity is considered a defensive cause for inguinal hernia. Contrarily, our study was inconsistent with the above-mentioned study.

BMI is the most accepted estimation of obesity. BMI lower than 18.5 kg/m² is considered underweight; between 18.5 and 24.99 kg/m² is considered to be the normal range; BMI exceeding 30 kg/m² is regarded as obese. Several studies have illustrated the effects of BMI on inguinal hernia incidence [15, 16]. Our study focused only on normal and low BMI, so those patients having normal BMI had chances of inguinal hernia either unilateral or bilateral.

The results of our study are following Sangwan et al. study, who reported a 76.35% incidence of inguinal hernia in Pakistan. Amongst 70% of patients of inguinal hernia, 92.20% were males, and 7.79% were females. Around 64.93% of inguinal hernias were found on the right side whilst 25.97% on the left side, and 9.09% of cases showed bilateral inguinal hernia [17].

The hernia occurrence depends upon risk factors, including gender, age, complaints of cough, repeated pregnancies, constipation, prior surgeries, lifting of heavy weights, obesity, genetic predisposition, and smoking [18]. A higher incidence of hernia among males (67.27%) than females (32.72%) was observed. Our study showed that males are more vulnerable to developing hernia than females - 78 (95.12%) males had an inguinal hernia, whereas four (4.87%) were females.

Similarly to other research such as by Gulzar et al. and Balram [19, 20], we found that most cases had a unilateral hernia compared to bilateral hernia. The bilateral hernia was observed only in 14 (100%) patients. Charles et al. also found that right-sided hernias are more frequent compared to left-sided [21]. This is also supported by our study - amongst unilateral hernias, the prevalence of the right side was slightly higher than in the left side with normal BMI 28 (82.4%) and 26 (76.5%), respectively.

Saeed et al. observed that 70% are right-sided, while 30% were left-sided, 46% indirect hernias, and 59% direct inguinal hernias [22]. Balram found that 62.3% of the inguinal hernias are on the right side than left or bilateral inguinal hernias [20]. Similar findings were observed in other studies; however, in our study, the unilateral right-sided inguinal hernia was more frequent than unilateral left-sided and bilateral inguinal hernia in patients with normal BMI. Some studies have proposed that hernia's right side dominance might be due to the late fall down of the right testis [23].

Velanovich et al. found a prevalence of hernia among 85% of males and 15% of females [24]. The present study also proved that males (n=78; 95.12%) were more affected than females (n=4; 4.87%) by an inguinal hernia, which was statistically insignificant (p=0.649).

In summary, the findings from this study help us to gain knowledge regarding the relationship between the incidence of IH and BMI. This study provides insight into the possible mechanisms behind the decrease in IH risk in obese patients, normal weight, or low weight with normal or low BMI. Limitations of our study include small sample size and confounding factors that could not be controlled by convenient sampling, for instance, the co-occurrence of other factors such as smoking, co-morbidities, and lifestyle. Based on our study results, we recommend more large-scale prospective studies in our population to explore the association of BMI with inguinal hernia.

## Conclusions

This study concluded that inguinal hernia is more prevalent in males than females, and it also proved a correlation between BMI and unilateral or bilateral inguinal hernia. We found that IH in both sides was significantly associated with normal BMI than low BMI; thus, we conclude that low BMI is a protective factor for IH development. Nevertheless, larger-scale studies are needed to support our conclusion. 
